# Factors influencing ankle alignment changes following medial unicompartmental knee arthroplasty: Preoperative knee and ankle deformities and extent of knee alignment correction

**DOI:** 10.1371/journal.pone.0318677

**Published:** 2025-03-14

**Authors:** Han-Ting Shih, Shun-Ping Wang, Cheng-Hung Lee, Kao-Chang Tu, Shih-Chieh Tang, Kun-Hui Chen

**Affiliations:** 1 Department of Orthopaedics, Taichung Veterans General Hospital, Taichung, Taiwan; 2 Department of Industrial Engineering and Enterprise Information, Tunghai University, Taichung, Taiwan; 3 Department of Post-Baccalaureate Medicine, College of Medicine, National Chung Hsing University, Taichung, Taiwan; 4 Department of Food Science and Technology, HungKuang University, Taichung, Taiwan; 5 Department of Computer Science and Information Engineering, Providence University, Taichung, Taiwan; Ningbo University, CHINA

## Abstract

**Introduction:**

The impact of medial unicompartmental knee arthroplasty (MUKA) on ankle alignment is not well-studied. This study aims to investigate the changes in ankle alignment following MUKA and identify the influencing factors.

**Materials and Methods:**

A retrospective analysis included 175 patients undergoing MUKA between 2018 and 2020. Patients were categorized into varus (n =  113) or valgus (n =  62) ankle groups based on preoperative ankle deformities. Preoperative and postoperative full-length standing radiographs were used for radiographic measurements.

**Results:**

Following MUKA, significant differences in the change in tibial plafond-talus angle (PTA) were observed between the groups, with the varus ankle group showing a change of -0.71 ±  0.82° and the valgus ankle group showing a change of 0.08 ±  0.94° (p <  0.001). In the varus ankle group, the tibial plafond-ground angle (PGA) increased from -3.65 ±  4.22° preoperatively to -0.51 ±  4.52° postoperatively (p <  0.001), talus-ground angle (TGA) increased from -5.28 ±  4.32° to -1.32 ±  4.74° (p <  0.001), and PTA decreased from 1.52 ±  1.04° to 0.81 ±  1.12° (p <  0.001). In the valgus ankle group, PGA increased from -5.44 ±  4.39° to -1.43 ±  4.63° (p <  0.001) and TGA increased from -4.55 ±  4.24° to -0.59 ±  4.47° (p <  0.001), but PTA did not show a significant change. Ankle alignment change significantly correlated with preoperative joint line convergence angle (JLCA), preoperative medial proximal tibial angle (MPTA), preoperative PGA, preoperative TGA, preoperative PTA, hip-knee-ankle angle (HKA) changes, and bearing thickness.

**Conclusions:**

MUKA significantly corrects the majority of ankle alignment towards a more neutral position. The extent of ankle alignment correction is influenced by preoperative knee and ankle joint deformities, as well as the degree of knee alignment correction.

## 1. Introduction

Medial unicompartmental knee arthroplasty (MUKA) is a widely accepted surgical option for treating osteoarthritis and osteonecrosis of the medial compartment of the knee [1]. By replacing only the damaged medial compartment with a prosthetic implant, MUKA preserves the lateral and patellofemoral compartments and the cruciate ligament, maintaining natural knee kinematics. Compared to total knee arthroplasty (TKA), MUKA offers smaller incisions, faster recovery, and better postoperative mobility [2]. This surgery has been shown to effectively alleviate knee joint symptoms, but it has been unexpectedly observed that some patients also experience changes in their ankle symptoms, whether worsening or improving [3]. Postoperative ankle symptoms subsequent to MUKA can lead to lower patient satisfaction and can affect postoperative clinical function and rehabilitation plans [3]. Therefore, investigating the impact of MUKA surgery on the ankle joint is an important issue to address.

Patients undergoing MUKA often present with medial compartment joint space narrowing and varus deformity of the knee joint. Given the close proximity of the knee and ankle joints, the alignment of these two joints can influence each other. Previous literature has also indicated that patients with knee joint deformities are more prone to experiencing malalignment in the ankle joint [4,5]. Ankle alignment refers to the spatial relationship between the tibial plafond and the talar dome, as well as their orientation relative to the ground. Although the relationship between ankle alignment and arthritis is not yet fully understood, it is evident that malalignment of the ankle joint can impact contact area and force transmission [6,7]. Surgeons must carefully assess ankle alignment before, during, and after MUKA surgery to prevent ankle joint complications and optimize surgical outcomes.

Correcting the alignment of the lower limb is a crucial goal in knee joint replacement surgery. However, just as knee deformities can affect ankle alignment, surgical interventions that alter knee alignment may simultaneously impact ankle alignment. Although numerous studies have investigated the influence of TKA on ankle alignment, there is a paucity of research focusing on the effects of MUKA on ankle alignment [8–10]. This gap in the literature is particularly notable given the unique biomechanical effects of MUKA, which preserves the lateral compartment and natural knee kinematics, potentially causing localized changes in ankle alignment distinct from TKA. These differences underscore the need for focused investigation. Furthermore, limited attention has been given to exploring the factors that contribute to changes in ankle alignment during MUKA surgery, particularly the impact of preoperative ankle joint deformity. Gaining a better understanding of these factors will provide surgeons with valuable insights into mitigating the occurrence of ankle malalignment during MUKA procedures.

The purpose of this study is to investigate the changes in ankle alignment following MUKA and analyze the influencing factors. Furthermore, it aims to investigate whether these changes vary across different preoperative ankle joint deformity conditions. The hypothesis is that the degree of change in ankle alignment is influenced by the following factors:

- Patient demographics
- Preoperative knee joint deformities
- Preoperative ankle joint deformities
- The extent of knee alignment correction
- The size and alignment of surgical components used

## 2. Materials and methods

### 2.1. Patient enrollment

This study complied with ethical standards and received approval from the Institutional Review Board (IRB) of Taichung Veterans General Hospital, with IRB approval number CE22468B. The IRB also approved the waiver of informed consent due to the retrospective nature of the study using extracted medical records data. The data for this research were accessed on May 30, 2024, for analysis purposes. Throughout the study, the authors did not have access to any information that could identify individual participants during or after data collection, ensuring patient anonymity. An a priori power analysis was conducted using G*Power 3 (Heinrich Heine Universität Düsseldorf, Germany) to determine the minimum required sample size for comparing changes in key radiographic measurements between two groups. Based on data from a similar study by Shih et al. [9], the effect size was calculated. The analysis estimated that a minimum of 64 participants would be required to detect a significant difference. The study enrolled patients who underwent MUKA between 2018 and 2020. Inclusion criteria required patients to have full-length standing anteroposterior radiographs both preoperatively and postoperatively. To minimize the influence of postoperative pain on standing posture and the potential changes in ankle alignment over an extended period, only patients who received full-length standing anteroposterior radiographs within 30 to 90 days postoperatively were included. Patients with prior surgery on the same limb, signs of extraarticular deformity, incomplete medical records, or inadequate imaging quality for analysis were excluded from the study. Four experienced joint replacement surgeons performed the surgeries included in this study. All implants used in the procedures were Oxford mobile bearing knee prostheses (Zimmer Biomet, Warsaw, IN, USA). A total of 175 patients were included in this study, with a mean age of 67.4 ±  7.6 years and a mean BMI of 28.2 ±  3.6 kg/m². Among them, 127 were female (72.6%) and 48 were male (27.4%). The mean follow-up duration for the patients was 44.6 ±  10.6 days. The demographic data for all patients are shown in Table 1.

**Table 1:** Demographic data and surgical characteristics of patients.

	Total (N = 175)		Varus ankle group (N = 113)		Valgus ankle group (N = 62)		*p* value ^a^
Age (years)	67.4	± 7.6	66.7	± 7.4	68.5	± 8.1	0.144
BMI (kg/m^2^)	28.2	± 3.6	28.5	± 3.6	27.7	± 3.4	0.160
Gender							0.077
Female	127	(72.6%)	87	(77.0%)	40	(64.5%)	
Male	48	(27.4%)	26	(23.0%)	22	(35.5%)	
Diagnosis							0.699
Osteoarthritis	168	(96.0%)	109	(96.5%)	59	(95.2%)	
Osteonecrosis	7	(4.0%)	4	(3.5%)	3	(4.8%)	
Side							0.068
Right	91	(52.0%)	53	(46.9%)	38	(61.3%)	
Left	84	(48.0%)	60	(53.1%)	24	(38.7%)	
Surgeon							0.809
Surgeon A	81	(46.3%)	51	(45.1%)	30	(48.4%)	
Surgeon B	49	(28.0%)	31	(27.4%)	18	(29.0%)	
Surgeon C	26	(14.9%)	19	(16.8%)	7	(11.3%)	
Surgeon D	19	(10.9%)	12	(10.6%)	7	(11.3%)	
Surgical approach							0.805
Subvastus	117	(66.9%)	77	(68.1%)	40	(64.5%)	
Midvastus	51	(29.1%)	32	(28.3%)	19	(30.6%)	
Medial parapatellar	7	(4.0%)	4	(3.5%)	3	(4.8%)	
Size of femoral component							0.116
Small	78	(44.6%)	56	(49.6%)	22	(35.5%)	
Extra small	50	(28.6%)	27	(23.9%)	23	(37.1%)	
Medium	40	(22.9%)	24	(21.2%)	16	(25.8%)	
Large	7	(4.0%)	6	(5.3%)	1	(1.6%)	
Size of tibial component							0.633
B	61	(34.9%)	42	(37.2%)	19	(30.6%)	
A	41	(23.4%)	25	(22.1%)	16	(25.8%)	
C	40	(22.9%)	23	(20.4%)	17	(27.4%)	
D	23	(13.1%)	17	(15.0%)	6	(9.7%)	
AA	10	(5.7%)	6	(5.3%)	4	(6.5%)	
Size of meniscal bearing							0.686
3 mm	105	(60.0%)	69	(61.1%)	36	(58.1%)	
4 mm	38	(21.7%)	23	(20.4%)	15	(24.2%)	
5 mm	24	(13.7%)	17	(15.0%)	7	(11.3%)	
6 mm	8	(4.6%)	4	(3.5%)	4	(6.5%)	

Categorical data were expressed as number (percentage). Continuous data are presented as mean ±  SD.

^a^Independent samples t-tests, chi-square test, or Fisher’s exact test.

### 2.2. Radiographic measurements

Full-length standing anteroposterior radiographs were obtained using a Computed Radiography (CR) system (Siemens Ysio Max, Siemens Healthineers, Germany) with standardized positioning and imaging parameters to ensure consistency and clarity. Due to detector size limitations, imaging was performed in three overlapping segments, followed by automated stitching and manual verification to prevent mismatches or distortion. All images were reviewed for quality assurance, with retakes performed as needed. Preoperative and postoperative full-length standing anteroposterior radiographs taken closest to the surgical date were used to measure parameters in knee and ankle joint. The parameters for the knee joint included the preoperative and postoperative hip-knee-ankle angle (HKA), the preoperative lateral distal femoral angle (LDFA), the preoperative medial proximal tibial angle (MPTA), and the preoperative joint line convergence angle (JLCA). The parameters for the ankle joint included the preoperative and postoperative tibial plafond-ground angle (PGA), talus-ground angle (TGA), and tibial plafond-talus angle (PTA). Additionally, both the femoral and tibial component alignments, including the femoral component alignment angle (FCA), tibial component alignment angle (TCA), and component vergence angle (CVA), were also measured. All measurements were conducted by a trained orthopedic surgeon, who was blinded to the study design, using the same Picture Archiving and Communication System (PACS) on a single computer. The measurer was required to perform two measurements and take their average to the first decimal place.

**Fig 1:**
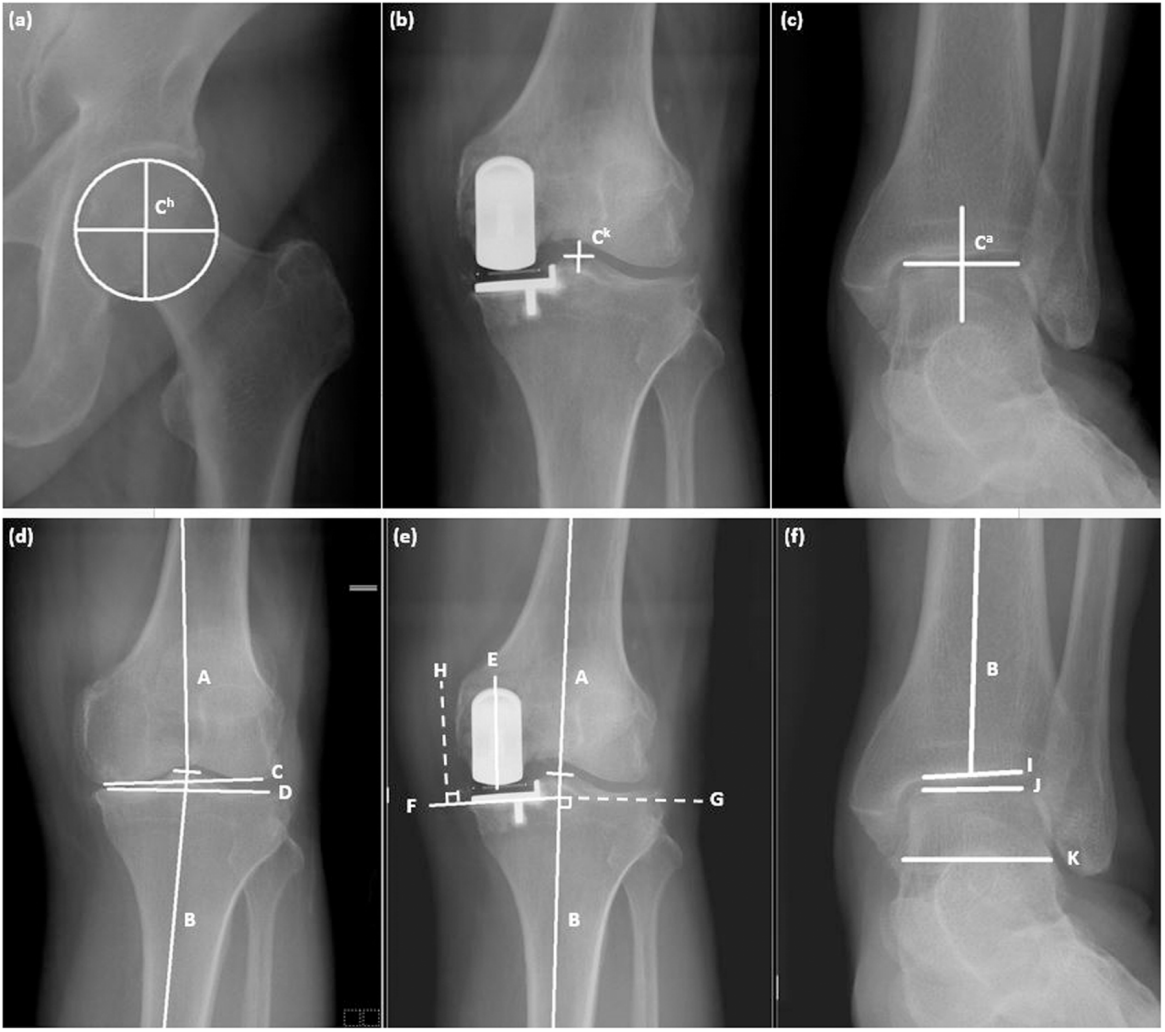
Radiographic measurements of knee and ankle joints. (a) The hip center (C^h^) is defined as the center of the femoral head. (b) The knee center (C^k^) is defined as the midpoint between the tibial spine tips. (c) The ankle center (C^a^) is defined as the midpoint of the articular surface of the talar dome. (d) The hip-knee-ankle angle (HKA) is defined as the angle between the mechanical axis of the femur (line A) and the mechanical axis of the tibia (line B). The lateral distal femoral angle (LDFA) is the lateral angle between line A and the joint line of the distal femur (line C). The medial proximal tibial angle (MPTA) is the medial angle between line B and the joint line of the proximal tibia (line D). The joint line convergence angle (JLCA) is the angle between line C and line D. (e) The femoral component alignment angle (FCA) is defined as the angle between line A and the longitudinal axis of the femoral component (line E). The tibial component alignment angle (TCA) is the angle between the undersurface of the tibial component (line F) and the vertical line to the mechanical axis of the tibia (line G). The component vergence angle (CVA) is the angle between line E and the vertical line to the undersurface of the tibial component (line H). (f) The tibial plafond-ground angle (PGA) is defined as the angle between the joint line of the tibial plafond (line I) and the ground line (line K). The talus-ground angle (TGA) is the angle between the joint line of the talar dome (line J) and line K. The tibial plafond-talus angle (PTA) is the angle between line I and line J.

The HKA is defined as the angle between the mechanical axis of the femur and the mechanical axis of the tibia. The mechanical axis of the femur is a line connecting the hip center, defined as the center of the femoral head, to the knee center, which is the midpoint of the tibial spine tips. [11]. The mechanical axis of the tibia is a line connecting the knee center to the ankle center, defined as the midpoint of the articular surface of the talar dome [12]. The LDFA is defined as the lateral angle between the mechanical axis of the femur and the joint line of the distal femur. The MPTA is defined as the medial angle between the mechanical axis of the tibia and the joint line of the proximal tibia. The JLCA is defined as the angle between the joint line of the distal femur and the joint line of the proximal tibia. The PGA and TGA are defined as the angles between the tibial plafond and the ground and between the talar dome and the ground, respectively. The PTA is defined as the angle between the tibial plafond and the talar dome [5]. The angle between the longitudinal axis of the femoral component and the mechanical axis of the femur was defined as the FCA [13–15]. Similarly, the angle between the undersurface of the tibial component and the perpendicular line of the mechanical axis of the tibia was defined as the TCA [14]. Additionally, the angle between the longitudinal axis of the femoral component and the perpendicular line of the undersurface of the tibial component was defined as the CVA [16]. The varus alignment of HKA, FCA, TCA, and CVA is defined as a positive value, while valgus alignment is defined as a negative value. The angles of JLCA, PGA, TGA, and PTA opening outward are defined as positive values, while those opening inward are defined as negative values. The difference between preoperative and postoperative parameters is represented by the symbol “ ∆ ”, including ∆HKA, ∆PGA, ∆TGA, and ∆PTA. All measurement methods are illustrated in Fig 1. According to preoperative ankle joint deformity, patients were categorized into varus ankle group and valgus ankle group, defined as preoperative PTA >  0° and preoperative PTA <  0°, respectively.

### 2.3. Statistic analysis

All statistical analyses were conducted using IBM SPSS Statistics for Windows, version 26 (IBM Corp., Armonk, NY, USA). The changes in knee and ankle alignment before and after the surgery were examined through paired sample t-tests. The relationship between ankle alignment changes and other parameters was analyzed using Pearson’s correlation coefficients. Differences in ankle alignment changes, demographic data, and surgical characteristics between groups were examined through independent samples t-tests, one-way analysis of variance, chi-square test, or Fisher’s exact test. Statistical significance was defined as p <  0.05 for all tests conducted in this study.

## 3. Results

### 3.1. Patient characteristics and grouping

The demographic data and surgical characteristics of all patients are presented in Table 1. The majority of surgeries were conducted on the right leg, comprising 91 patients (52.0%). Osteoarthritis was the primary indication for surgery, with 168 patients (96.0%) undergoing treatment for this condition. According to preoperative ankle joint deformity, 113 patients were categorized into the varus ankle group, and 62 patients were categorized into the valgus ankle group. There were no significant differences in demographic data and surgical characteristics between the two groups (Table 1). In terms of radiologic results, significant differences were observed in preoperative PGA (p =  0.009), preoperative PTA (p <  0.001), postoperative PTA (p <  0.001), and ∆ PTA (p <  0.001) between the two groups (Table 2).

**Table 2:** Radiologic results of patients.

	Total (N = 175)		Varus ankle group (N = 113)		Valgus ankle group (N = 62)		*p* value ^a^
Preoperative alignment							
HKA (°)	9.47	± 4.22	9.57	± 3.77	9.31	± 4.97	0.724
LDFA (°)	89.05	± 2.19	89.04	± 1.94	89.08	± 2.61	0.909
MPTA (°)	84.21	± 2.87	84.05	± 2.86	84.52	± 2.88	0.298
JLCA (°)	4.59	± 2.62	4.52	± 2.51	4.73	± 2.82	0.601
PGA (°)	-4.29	± 4.35	-3.65	± 4.22	-5.44	± 4.39	0.009 *
TGA (°)	-5.02	± 4.29	-5.28	± 4.32	-4.55	± 4.24	0.281
PTA (°)	0.65	± 1.54	1.52	± 1.04	-0.95	± 0.90	< 0.001**
Postoperative alignment							
HKA (°)	3.61	± 3.71	3.57	± 3.50	3.70	± 4.10	0.815
PGA (°)	-0.84	± 4.57	-0.51	± 4.52	-1.43	± 4.63	0.205
TGA (°)	-1.06	± 4.65	-1.32	± 4.74	-0.59	± 4.47	0.316
PTA (°)	0.22	± 1.39	0.81	± 1.12	-0.87	± 1.17	< 0.001**
FCA (°)	0.77	± 4.19	0.62	± 4.12	1.05	± 4.35	0.518
TCA (°)	4.97	± 3.21	4.94	± 3.35	5.02	± 2.97	0.876
CVA (°)	2.12	± 5.64	2.03	± 5.42	2.28	± 6.07	0.775
Difference (∆)							
HKA (°)	-5.86	± 3.09	-6.00	± 3.07	-5.61	± 3.12	0.422
PGA (°)	3.45	± 3.91	3.14	± 3.97	4.01	± 3.76	0.161
TGA (°)	3.96	± 3.75	3.95	± 3.73	3.96	± 3.81	0.994
PTA (°)	-0.43	± 0.94	-0.71	± 0.82	0.08	± 0.94	< 0.001**

HKA hip-knee-ankle angle; LDFA lateral distal femoral angle; MPTA medial proximal tibial angle; JLCA joint line convergence angle; PGA tibial plafond-ground angle; TGA talus-ground angle; PTA tibial plafond-talus angle; FCA femoral component alignment angle; TCA tibial component alignment angle, CVA component vergence angle.

^a^Independent samples t-tests. ^* ^ p <  0.05, ^**^ p <  0.01.

### 3.2. Radiologic results for all patients

**Fig 2:**
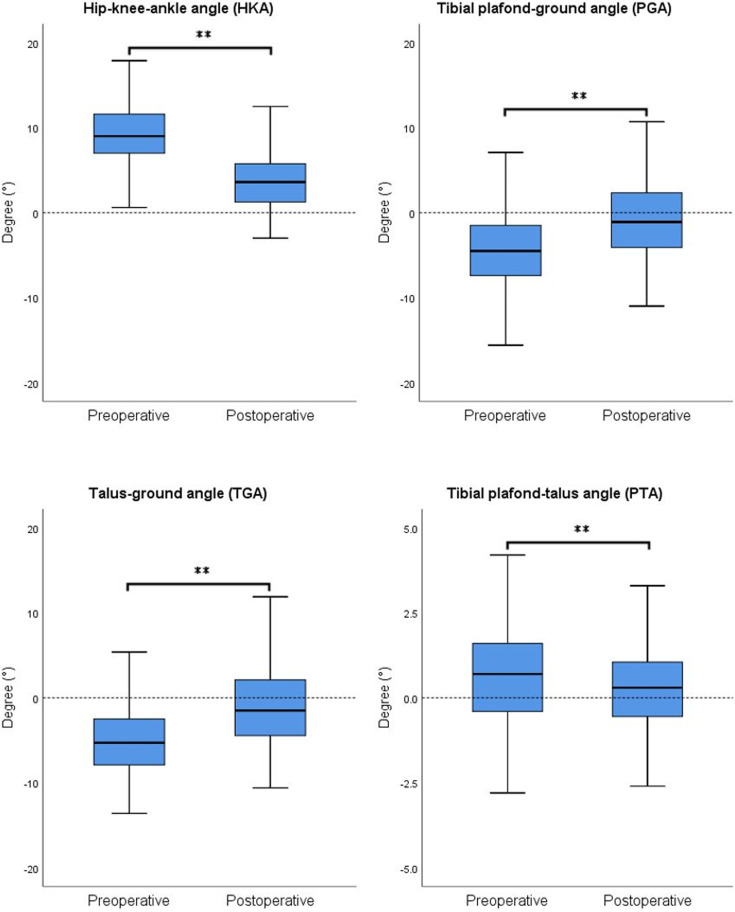
Radiographic results for all patients. Both knee and ankle alignments were significantly corrected to a relative neutral position following MUKA. Analysis was performed using paired sample t-tests for preoperative and postoperative comparisons. ** represents p <  0.01.

The radiographic results for all patients are presented in Table 2. Both the alignment of the knee joint and the ankle joint showed significant changes after the surgery (Fig 2). For the knee joint, the average preoperative HKA was 9.47 ±  4.22°, which significantly decreased to 3.61 ±  3.71° postoperatively (p <  0.001). Regarding the ankle joint, the average preoperative PGA was -4.29 ±  4.35°, which significantly increased to -0.84 ±  4.57° postoperatively (p <  0.001). Similarly, the average preoperative TGA was -5.02 ±  4.29°, which significantly increased to -1.06 ±  4.65° postoperatively (p <  0.001). Additionally, the average preoperative PTA was 0.65 ±  1.54°, and it significantly decreased to 0.22 ±  1.39° postoperatively (p <  0.001).

In the correlation analysis, both ∆PGA and ∆TGA were significantly correlated with preoperative JLCA, preoperative PGA, preoperative TGA, ∆HKA, and bearing thickness. The correlations with ∆PGA were as follows: preoperative JLCA (r =  0.240, p =  0.001), preoperative PGA (r =  -0.393, p <  0.001), preoperative TGA (r =  -0.317, p <  0.001), ∆HKA (r =  -0.361, p <  0.001), and bearing thickness (r =  0.225, p =  0.003). Similarly, the correlations with ∆TGA were: preoperative JLCA (r =  0.209, p =  0.005), preoperative PGA (r =  -0.330, p <  0.001), preoperative TGA (r =  -0.338, p <  0.001), ∆HKA (r =  -0.359, p <  0.001), and bearing thickness (r =  0.224, p =  0.003). On the other hand, ∆PTA was only correlated with preoperative MPTA (r =  0.227, p =  0.002) and preoperative PTA (r =  -0.456, p <  0.001) (Table 3).

There were no significant differences in ∆PGA, ∆TGA, and ∆PTA among different groups based on gender, side, diagnosis, surgeon, surgical approach, femoral component size, or tibial component size.

**Table 3:** Correlation between ankle joint alignment changes and parameters for all patients.

	∆PGA		∆TGA		∆PTA	
	*r*	*p* value ^a,^	*r*	*p* value ^a^	*r*	*p* value ^a^
Age	0.027	0.725	0.012	0.879	0.092	0.228
BMI	0.137	0.071	0.126	0.096	0.004	0.956
Preoperative HKA	0.062	0.413	0.090	0.234	-0.106	0.164
Preoperative LDFA	-0.027	0.726	0.012	0.872	-0.083	0.273
Preoperative MPTA	0.087	0.250	0.047	0.537	0.227	0.002**
Preoperative JCLA	0.240	0.001**	0.209	0.005**	0.144	0.058
Preoperative PGA	-0.393	< 0.001**	-0.330	< 0.001**	-0.143	0.059
Preoperative TGA	-0.317	< 0.001**	-0.338	< 0.001**	-0.010	0.895
Preoperative PTA	-0.102	0.180	0.007	0.923	-0.456	< 0.001**
FCA	-0.063	0.405	-0.023	0.761	-0.086	0.259
TCA	-0.146	0.054	-0.125	0.099	0.009	0.905
CVA	0.027	0.721	0.046	0.550	0.021	0.786
∆HKA	-0.361	< 0.001**	-0.359	< 0.001**	0.013	0.867
Bearing thickness	0.225	0.003**	0.224	0.003**	-0.047	0.537

HKA hip-knee-ankle angle; LDFA lateral distal femoral angle; MPTA medial proximal tibial angle; JLCA joint line convergence angle; PGA tibial plafond-ground angle; TGA talus-ground angle; PTA tibial plafond-talus angle; FCA femoral component alignment angle; TCA tibial component alignment angle, CVA component vergence angle.

^a^Pearson’s correlation coefficients. ^* ^ p <  0.05, ^**^ p <  0.01.

### 3.3. Radiologic results for patients in varus ankle group

**Fig 3:**
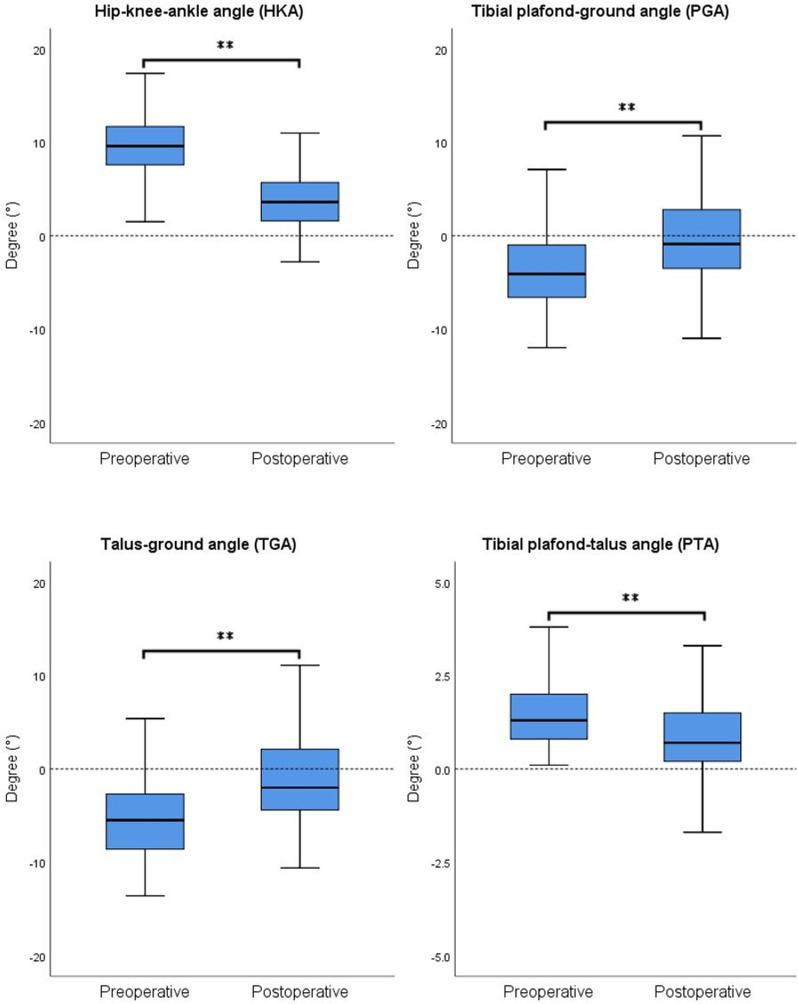
Radiographic results for patients in varus ankle group. Both knee and ankle alignments were significantly corrected to a relative neutral position following MUKA. Analysis was performed using paired sample t-tests for preoperative and postoperative comparisons. ** represents p <  0.01.

In the varus ankle group, both knee joint and ankle joint alignment exhibited significant changes after the surgery (Fig 3). For the knee joint, the average preoperative HKA angle was 9.57 ±  3.77°, significantly decreasing to 3.57 ±  3.50° postoperatively (p <  0.001). Concerning the ankle joint, the average preoperative PGA was -3.65 ±  4.22°, significantly increasing to -0.51 ±  4.52° postoperatively (p <  0.001). The average preoperative TGA was -5.28 ±  4.32°, significantly increasing to -1.32 ±  4.74° postoperatively (p <  0.001). Additionally, the average preoperative PTA was 1.52 ±  1.04°, and it significantly decreased to 0.81 ±  1.12° postoperatively (p <  0.001). In the correlation analysis, ∆PGA showed significant correlations with preoperative JCLA (r =  0.200, p =  0.034), preoperative PGA (r =  -0.391, p <  0.001), preoperative TGA (r =  -0.311, p =  0.001), ∆HKA (r =  -0.354, p <  0.001), and bearing thickness (r =  0.203, p =  0.031). ∆TGA showed significant correlations with preoperative PGA (r =  -0.317, p =  0.001), preoperative TGA (r =  -0.314, p =  0.001), ∆HKA (r =  -0.343, p <  0.001), and bearing thickness (r =  0.209, p =  0.026). ∆PTA showed significant correlations with preoperative JCLA (r =  0.196, p =  0.037), and preoperative PTA (r =  -0.289, p =  0.002) (Table 4). No significant differences in ankle alignment changes were observed among different groups based on patient demographics and surgical characteristics.

**Table 4:** Correlation between ankle joint alignment changes and parameters for patients in varus ankle group.

	∆PGA		∆TGA		∆PTA	
	*r*	*p* value ^a^	*r*	*p* value ^a^	*r*	*p* value ^a^
Age	0.105	0.270	0.115	0.226	0.027	0.774
BMI	0.142	0.133	0.117	0.217	0.028	0.770
Preoperative HKA	0.018	0.851	0.033	0.728	-0.035	0.711
Preoperative LDFA	-0.015	0.877	0.044	0.647	-0.081	0.393
Preoperative MPTA	0.118	0.214	0.105	0.270	0.177	0.061
Preoperative JCLA	0.200	0.034 *	0.166	0.079	0.196	0.037 *
Preoperative PGA	-0.391	< 0.001**	-0.317	0.001**	-0.175	0.064
Preoperative TGA	-0.311	0.001**	-0.314	0.001**	-0.156	0.098
Preoperative PTA	-0.034	0.723	0.010	0.918	-0.289	0.002**
FCA	-0.045	0.637	0.003	0.978	-0.031	0.741
TCA	-0.021	0.824	-0.001	0.988	0.078	0.412
CVA	0.145	0.127	0.173	0.067	0.087	0.357
∆HKA	-0.354	< 0.001**	-0.343	< 0.001**	-0.029	0.761
Bearing thickness	0.203	0.031 *	0.209	0.026 *	-0.064	0.501

HKA hip-knee-ankle angle; LDFA lateral distal femoral angle; MPTA medial proximal tibial angle; JLCA joint line convergence angle; PGA tibial plafond-ground angle; TGA talus-ground angle; PTA tibial plafond-talus angle; FCA femoral component alignment angle; TCA tibial component alignment angle, CVA component vergence angle.

^a^Pearson’s correlation coefficients. ^* ^ p <  0.05, ^**^ p <  0.01.

### 3.4. Radiologic results for patients in valgus ankle group

**Fig 4:**
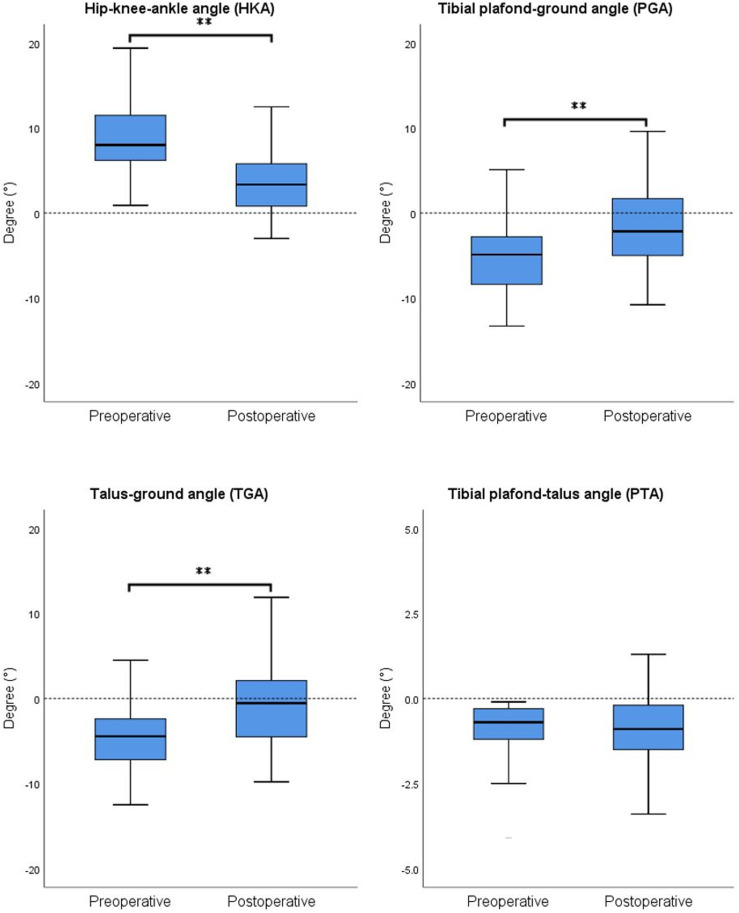
Radiographic results for patients in valgus ankle group. Knee and ankle alignments, including HKA, PGA, TGA, were significantly corrected to a relatively neutral position following MUKA. However, there were no significant differences in PTA between preoperative and postoperative measurements. Analysis was performed using paired sample t-tests for preoperative and postoperative comparisons. ** represents **p** <  0.01.

In the valgus ankle group, HKA, PGA, and TGA all showed significant changes after the surgery, whereas PTA did not exhibit a statistically significant change (Fig 4). The average HKA decreased from 9.31 ±  4.97° preoperatively to 3.70 ±  4.10° postoperatively (p <  0.001). The average PGA increased from -5.44 ±  4.39° preoperatively to -1.43 ±  4.63° postoperatively (p <  0.001). The average TGA increased from -4.55 ±  4.24° preoperatively to -0.59 ±  4.47° postoperatively (p <  0.001). In the correlation analysis, ∆PGA exhibited significant correlations with preoperative JCLA (r =  0.305, p =  0.016), preoperative PGA (r =  -0.364, p =  0.004), preoperative TGA (r =  -0.365, p =  0.004), ∆HKA (r =  -0.402, p =  0.001), and bearing thickness (r =  0.262, p =  0.040). ∆TGA showed significant correlations with preoperative JCLA (r =  0.280, p =  0.028), preoperative PGA (r =  -0.371, p =  0.003), preoperative TGA (r =  -0.385, p =  0.002), and ∆HKA (r =  -0.389, p =  0.002). ∆PTA showed significant correlations with preoperative MPTA (r =  0.275, p =  0.030) (Table 5). No significant differences in ankle alignment changes were observed among different groups based on patient demographics and surgical characteristics.

**Table 5:** Correlation between ankle joint alignment changes and parameters for patients in valgus ankle group.

	∆PGA		∆TGA		∆PTA	
	*r*	*p* value ^a^	*r*	*p* value ^a^	*r*	*p* value ^a^
Age	-0.143	0.267	-0.158	0.221	0.088	0.498
BMI	0.165	0.200	0.147	0.255	0.094	0.466
Preoperative HKA	0.139	0.283	0.171	0.184	-0.186	0.148
Preoperative LDFA	-0.048	0.712	-0.029	0.821	-0.113	0.383
Preoperative MPTA	0.006	0.962	-0.056	0.667	0.275	0.030 *
Preoperative JCLA	0.305	0.016 *	0.280	0.028 *	0.058	0.654
Preoperative PGA	-0.364	0.004**	-0.371	0.003**	0.089	0.493
Preoperative TGA	-0.365	0.004**	-0.385	0.002**	0.132	0.306
Preoperative PTA	-0.026	0.844	0.018	0.888	-0.186	0.147
FCA	-0.114	0.378	-0.067	0.603	-0.244	0.056
TCA	-0.170	0.130	-0.137	0.223	-0.129	0.319
CVA	-0.182	0.156	-0.159	0.217	-0.094	0.466
∆HKA	-0.402	0.001**	-0.389	0.002**	0.012	0.925
Bearing thickness	0.262	0.040 *	0.249	0.051	-0.063	0.628

HKA hip-knee-ankle angle; LDFA lateral distal femoral angle; MPTA medial proximal tibial angle; JLCA joint line convergence angle; PGA tibial plafond-ground angle; TGA talus-ground angle; PTA tibial plafond-talus angle; FCA femoral component alignment angle; TCA tibial component alignment angle, CVA component vergence angle.

^a^Pearson’s correlation coefficients. *  p <  0.05, ** p <  0.01.

## 4. Discussion

**Fig 5:**
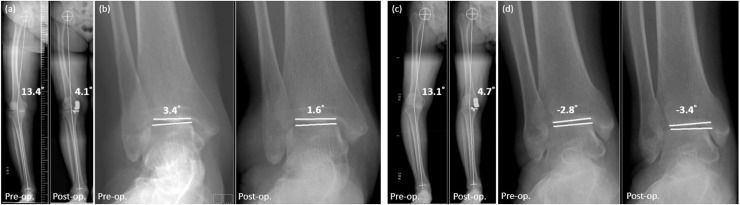
Ankle alignment changes following MUKA and its association with preoperative ankle deformities. While preoperative varus ankle deformity is significantly corrected postoperatively, there is limited improvement in cases of preoperative valgus ankle deformity. (a) Patients with preoperative varus ankle deformity exhibited a correction in HKA angle from 13.4° to 4.1°, and (b) a reduction in PTA angle from 3.4° to 1.6°. (c) Patients with preoperative valgus ankle deformity showed a correction in HKA angle from 13.1° to 4.7°, while (d) the PTA angle changed from -2.8° to -3.4°.

The main finding of this study was that most of the ankle alignments were corrected to a relatively neutral position following MUKA. The extent of ankle joint correction was found to be associated with the preoperative deformity of the knee and ankle joints, as well as the extent of knee joint correction. However, differences in alterations to ankle alignment induced by MUKA were noted, depending on the specific preoperative deformities present in the ankle joint (Fig 5). These findings underscore the clinical importance of understanding the relationship between knee and ankle alignment to optimize surgical outcomes and address patient-specific deformities. Achieving a neutral ankle alignment postoperatively optimizes load distribution and joint stability, which may explain why some patients experience alleviation of ankle symptoms after surgery. Conversely, residual malalignment may lead to functional limitations and difficulties in achieving full recovery, ultimately resulting in lower postoperative satisfaction. For surgeons, these findings emphasize the importance of comprehensive care across all stages of treatment. Preoperatively, a detailed assessment of ankle joint alignment and existing deformities is crucial for surgical planning. Intraoperatively, achieving precise alignment is vital to reducing residual deformities that could hinder recovery. Postoperatively, tailored rehabilitation is crucial for addressing residual malalignment and optimizing outcomes after MUKA. Recent research underscores the importance of optimizing movement strategies to reduce lower limb injury risks, highlighting the need for biomechanically informed rehabilitation approaches [17]. Addressing these factors holistically can enhance surgical and rehabilitative strategies, improving patient care and satisfaction.

An increasing number of studies have focused on investigating the interactions between adjacent joints, with one of the most prominent examples being the spinopelvic relationship, which may contribute to issues like hip dislocation [18]. Similarly, the relationship between the knee and ankle joints has gained attention due to its clinical and biomechanical significance. Biomechanical analyses have shown that alterations in joint alignment can significantly influence load distribution and dynamic stability, as highlighted in recent studies exploring joint compensation and force transmission mechanisms [19]. However, the literature on ankle alignment changes following MUKA remains limited [3,20]. In the study conducted by Sari et al., changes were observed in all ankle alignment parameters, including PGA, TGA, and PTA. However, the authors did not statistically validate the significant changes that occurred [20]. This lack of statistical validation contrasts with the present analysis, which provides robust evidence for these alignment changes, enhancing the reliability of the findings. Similarly, Güngör et al. reported similar results to the current study, demonstrating significant correction in TGA and PTA after MUKA, but no analysis was conducted for PGA [3]. This study expands upon these findings by integrating a broader range of ankle parameters, providing a more holistic assessment of alignment changes and emphasizing the importance of patient-specific surgical planning to optimize biomechanical outcomes. Furthermore, these previous studies did not analyze factors that may influence changes in ankle alignment. This research emphasizes the interplay between preoperative knee deformities, the extent of knee correction, and subsequent ankle alignment adjustments, highlighting the biomechanical relationship of adjacent joints. To the best of our knowledge, this study is the first to compare changes in ankle alignment after MUKA in different preoperative ankle deformities and investigate the relationship between knee alignment correction and preoperative lower limb deformities concerning ankle alignment changes.

The correlational analysis results in this study demonstrate that larger preoperative deformities in both the knee and ankle joints and a greater degree of correction in knee joint alignment are both associated with a more significant extent of ankle alignment correction. The preoperative knee alignment, preoperative ankle alignment, intraoperative changes in knee alignment, and intraoperative changes in ankle alignment are four parameters that directly or indirectly influence each other. Patients with larger preoperative knee joint deformities often exhibit larger preoperative ankle joint deformities [4,5,21,22]. Additionally, greater preoperative joint deformity indicates a greater potential for joint alignment correction [23]. MUKA corrects the alignment of the knee joint, resulting in a more vertical orientation of the tibia compared to the preoperative state. Simultaneously, it aligns the joint line of the tibia plafond and talus to a more horizontal orientation. On the other hand, changes in knee alignment are closely related to the thickness of the bearing. Thicker bearing thickness results in a greater correction of the HKA [24,25]. The impact of these influencing factors has been extensively documented in TKA; however, it is the first time they have been identified in MUKA [9,26–28]. These findings can assist surgeons in accurately predicting postoperative ankle alignment and help prevent the occurrence of ankle malalignment or symptoms.

In this study, significant differences in ankle alignment correction were observed between the varus ankle group and valgus ankle group, particularly in the change of the tibial plafond-talus angle. Preoperative ankle deformities tend to shift towards more valgus alignment after surgery if they were initially aligned in varus, and conversely, towards more varus alignment if initially aligned in valgus. After MUKA, both groups tend to achieve a more neutral alignment in the ankle joint. Moreover, while preoperative varus ankle deformity is significantly corrected postoperatively, there is limited improvement in cases of preoperative valgus ankle deformity. These discrepancies may be attributed to the varying compensatory capabilities of the subtalar joint and fixed ankle deformities, factors that were beyond the scope of this study. The subtalar joint plays a crucial role in maintaining lower limb alignment by redistributing loads and adjusting through inversion or eversion to compensate for malalignment [29]. However, subtalar joint compensation is not guaranteed to occur, and its capacity to compensate may be influenced by adjacent joint conditions, particularly those of the ankle joint [9,30]. The interplay between the subtalar joint and ankle joint is intricately complex. When there is malalignment or a change in alignment in the lower extremities, the compensatory effects of the subtalar joint can impact alterations in ankle alignment [27,31]. Conversely, these compensatory effects are also subject to the type and severity of deformities present in the ankle joint [32,33]. Similar phenomena have been observed in other surgeries that alter lower limb alignment. In TKA, different preoperative ankle joint deformities can result in varying degrees of correction and even impact the occurrence of postoperative ankle joint degeneration [9,30]. On the other hand, as a retrospective study, comprehensive ankle and foot alignment as well as symptoms could not be documented, leading to some fixed ankle deformities, such as posttraumatic arthritis and osteoarthritis, remaining undiagnosed preoperatively. Future research should aim to investigate the complex interplay between the subtalar and ankle joints using prospective designs with more detailed evaluations, including advanced imaging and functional assessments, to provide further insights.

This study has several limitations that need to be considered. Firstly, as a retrospective study, selection bias and confounding factors, such as activity levels, may affect results. Activity levels influence posture during imaging and recovery, impacting measurement accuracy and alignment adaptation. Standardized imaging and activity assessments in future studies could address these issues. Factors such as ligamentous laxity or compensatory changes in the subtalar joint, which could potentially influence ankle alignment, were not investigated. Incorporating stress radiographs or advanced imaging in future studies may provide insights into these influences. Secondly, the short follow-up duration in this study limits the findings to immediate postoperative changes in ankle alignment. Over time, mechanical forces, bone remodeling, and soft tissue adaptation may progressively alter joint alignment. Additionally, factors such as implant wear, patient activity, and weight changes could further impact these parameters. These potential long-term changes highlight the need for future studies incorporating longitudinal analyses to better understand the evolution of ankle alignment. Thirdly, only radiographic measurements were utilized to assess ankle alignment, and the clinical implications of these alignment changes were not evaluated. Including clinical outcome measures in future studies could help link radiographic changes to patient satisfaction and function. Fourthly, all measurements were derived from full-length standing anteroposterior radiographs, which may introduce errors in cases of lower limb rotation or flexion contracture. Additionally, the measurement of lateral view parameters and the assessment of subtalar joint alignment were not possible. Weight-bearing CT or other multi-planar imaging techniques could address these limitations.

## 5. Conclusions

In conclusion, this study demonstrated that MUKA surgery resulted in significant correction of a majority of ankle alignments, effectively bringing them closer to a neutral position. The degree of ankle joint correction was influenced by preoperative knee and ankle joint deformities, as well as the extent of knee alignment correction achieved during surgery. These findings highlight the importance of assessing and addressing ankle alignment in MUKA procedures to optimize surgical outcomes and minimize the risk of postoperative ankle joint complications. Further prospective studies are warranted to explore additional factors and clinical implications associated with ankle alignment changes following MUKA surgery.

## Supporting information

S1 Data Anonymized data used for analysis. (XLSX)
